# Network Based Integrated Analysis of Phenotype-Genotype Data for Prioritization of Candidate Symptom Genes

**DOI:** 10.1155/2014/435853

**Published:** 2014-06-02

**Authors:** Xing Li, Xuezhong Zhou, Yonghong Peng, Baoyan Liu, Runshun Zhang, Jingqing Hu, Jian Yu, Caiyan Jia, Changkai Sun

**Affiliations:** ^1^School of Computer and Information Technology and Beijing Key Lab of Traffic Data Analysis and Mining, Beijing Jiaotong University, Beijing 100044, China; ^2^School of Engineering and Informatics, University of Bradford, West Yorkshire BD7 1DP, UK; ^3^China Academy of Chinese Medical Sciences, Beijing 100700, China; ^4^Guang'anmen Hospital, China Academy of Chinese Medical Sciences, Beijing 100053, China; ^5^Institute of Basic Theory of Traditional Chinese Medicine, China Academy of Chinese Medical Sciences, Beijing 100700, China; ^6^Liaoning Provincial Key Laboratory of Cerebral Diseases, Institute for Brain Disorders, Dalian Medical University, Dalian 116044, China

## Abstract

*Background*. Symptoms and signs (symptoms in brief) are the essential clinical manifestations for individualized diagnosis and treatment in traditional Chinese medicine (TCM). To gain insights into the molecular mechanism of symptoms, we develop a computational approach to identify the candidate genes of symptoms. * Methods*. This paper presents a network-based approach for the integrated analysis of multiple phenotype-genotype data sources and the prediction of the prioritizing genes for the associated symptoms. The method first calculates the similarities between symptoms and diseases based on the symptom-disease relationships retrieved from the PubMed bibliographic database. Then the disease-gene associations and protein-protein interactions are utilized to construct a phenotype-genotype network. The PRINCE algorithm is finally used to rank the potential genes for the associated symptoms. * Results*. The proposed method gets reliable gene rank list with AUC (area under curve) 0.616 in classification. Some novel genes like CALCA, ESR1, and MTHFR were predicted to be associated with headache symptoms, which are not recorded in the benchmark data set, but have been reported in recent published literatures. * Conclusions*. Our study demonstrated that by integrating phenotype-genotype relationships into a complex network framework it provides an effective approach to identify candidate genes of symptoms.

## 1. Introduction

Traditional Chinese medicine (TCM) is an essential part of the healthcare system in China. TCM diagnosis and treatment are formed based on a comprehensive analysis of the clinical manifestations obtained through four main procedures: observation, listening, questioning, and pulse analysis [1]. Patients with different diseases would often manifest different symptoms and signs, such as anorexia and pain, which are the evidences to be considered by physicians for clinical diagnoses in TCM [2].

Although symptoms play important role in modern biomedical diagnosis and disease classification, most modern biomedical research attempts to gain understanding of the molecular mechanism of disease phenotypes [3], including investigating the genotypes of disease/disease categories. Likewise, in the TCM field, attempt has also been made to investigate the genotypes or molecular mechanisms of the diagnosis (i.e., TCM syndrome) [4, 5].

A recent research showed that there exist metabolic biomarkers of clinical manifestations like symptoms and syndromes in different types of rheumatoid arthritis (RA) diseases [6]. However, there is no clear understanding of the underlying molecular mechanism of symptoms and the principle of TCM syndrome in TCM field.

Large-scale diagnosis and phenotype-genotype association data, including both published literature and manually curated databases, have been gathered in the last decades [7]. PubMed, which is a public-available biomedical bibliographic database, provides a significant resource for studying the associations between diseases and clinical manifestations [8]. The phenotype-genotype association database like OMIM [9] contains high-quality data on relationships between diseases and genes. In addition, large-scale molecular network data are available [10–12], such as protein-protein interaction data, metabolic pathway data, and gene regulation data. Those provide important resources to explore the molecular correlations of symptoms.

In this paper, we first extracted the symptom-disease relationships from PubMed bibliographic records. We used the cosine similarities to evaluate the association between symptoms and diseases. We then integrated the symptom-disease relationships with disease-gene associations and protein-protein interactions (PPI) to construct a new database recording the associations between symptoms and genes. We finally used the PRINCE algorithm to rank the potential genes of symptoms. We evaluate the results of the prediction by using manually curated symptom-gene data set and PubMed literature searching. The evaluation shows that the results suggest medical meaningful insight.

## 2. Related Work

Using network-based approaches to gain insights into human disease has found multiple potential biological and clinical applications [13]. Further understanding of the effects of cellular interconnectedness on disease progression leads to the identification of disease biomarker genes and the pathways causing the associated diseases [14], which, in turn, offer effective targets for new drug development. Many human genetic diseases are caused by multiple genes. For genes that are associated with the same or similar phenotypes, the genes are likely to be functionally related. Such relations can be exploited to aid in searching for novel disease genes. Computational approaches have recently been proposed to predict associations between genes and diseases [15–17]. Vanunu et al. developed a network-based approach, which is known as PRINCE algorithm, for predicting causal genes and protein complexes involved in a disease of interest [18]. The availability of large-scale data of phenotype-genotype associations like OMIM, CTD [19], and PharmGKB [20] provides valuable resources for studying disease-gene associations.

Recently increasing interest on the study of molecular mechanism of symptoms was found. The underlying molecular mechanisms of several symptoms, such as depression, pain, and high blood pressure, have been discussed previously [21–23]. However, no work has been done to investigate systematically the mechanism of symptoms in the literature. Until recently, Zhou et al. used large-scale biomedical literature database to construct a symptom-based human disease network and investigate the associations between clinical manifestations of diseases and the underlying molecular interactions [24]. Their results showed that symptom-based similarity of diseases correlates strongly with the number of shared genetic associations and the extent to which their associated proteins interact. This indicates that symptoms would have their underlying molecular mechanisms needed to be further explored. In this paper, we attempt to develop a new data mining framework to explore the relationships between symptoms and genes, which may provide scientific evidences to traditional Chinese medicine in individualized diagnosis and treatment because symptoms are the main clinical manifestations captured by TCM physicians for both diagnosis and treatment.

## 3. Methods

### 3.1. Phenotype-Genotype Data Integration

In order to extract the associations between symptoms and genes, we first built symptom-disease associations based on a large number of medical literatures in PubMed [25] and the Medical Subject Headings (MeSH). Using the cooccurrence of diseases and symptoms, we construct two vectors *s* and *d* to calculate the similarity of symptom and disease, in which *d* denotes a disease vector represented by its cooccurrence symptoms and *s* denotes a symptom vector represented by its cooccurrence symptoms as well. Suppose we have a dictionary with *n* symptom items, we would have an *n*-features vector for both disease and symptom. Based on the vectors of diseases and symptoms, we calculate the similarity of symptom and disease using cosine correlation:

(1) $$\begin{array}{c} T\left(d,s\right)=\frac{d\cdot s}{{||d||}^{2}\times {||s||}^{2}}. \end{array}$$

**Figure 1:**
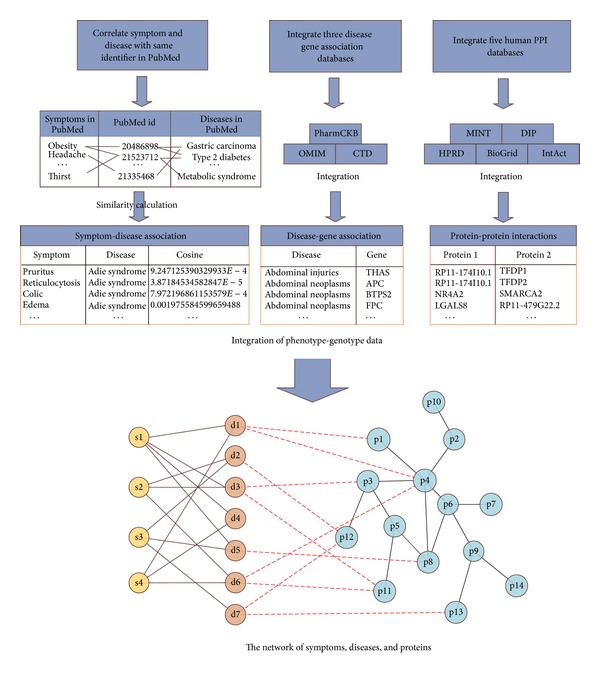
The integration of phenotype-genotype data. Symptom-disease associations are extracted based on the fact that the symptom and disease appeared in same bibliographic record (including title, abstract, and MeSH) of PubMed. Three disease gene association databases (i.e., OMIM, CTD, and PharmGKB) and five human PPI databases (i.e., HPRD, BioGrid, IntAct, MINT, and DIP) are integarted in this study. The relationships among symptoms (denoted s1–s4), diseases (denoted d1–d7), and proteins (denoted p1–p14) are then extracted.

In this study, we integrated three public available disease-gene databases (OMIM, CTD, and PharmGKB) and five protein-protein interactions databases (HPRD, BioGrid, IntAct, MINT, and DIP) into database (Figure 1). Based on these data sets a heterogeneous network is constructed with nodes representing symptoms, diseases, and proteins, respectively, and the links representing symptom-disease relationships, disease-gene associations, and protein-protein interactions.

### 3.2. Network Inference for Prioritization of Symptom Candidate Genes

**Figure 2:**
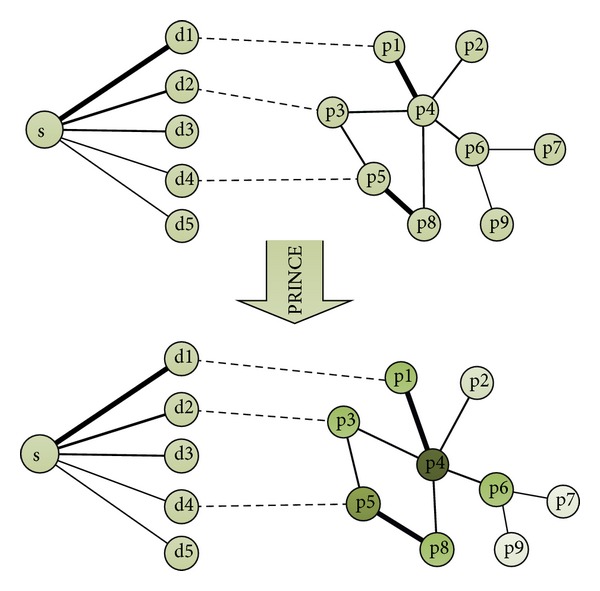
The approach for predicting the genes with respect to symptom using PRINCE algorithm. For a query symptom S, it has varying degrees of relationship with other diseases, denoted by d1–d5 (where the thickness of lines represents degree of correlation between symptom and diseases). p1–p9 comprise the protein set of a protein-protein interaction network, where interactions are denoted by lines with different thickness (confidence). PRINCE uses an iterative propagation method to assign a score of each protein. The protein with higher score is considered to be the causal gene candidate for symptom S.

The network-based disease gene prediction approach, PRINCE, is used for predicting the genes with respect to symptom. The initialization of the parameters in PRINCE algorithm is the symptom-disease correlations, disease-gene associations, and protein-protein interactions. It uses a propagation-based algorithm [26] to infer a scoring function for estimating the strength of an association. A score is defined for each gene, which reflects the prior information of the genes on the related disease. The score is then used in combination with a PPI network for the identification of proteins involved in the given symptom, as shown in Figure 2. 

### 3.3. Computing the Prioritization Function

The prioritization of genes for a query symptom (*s*) is performed based on the given symptom-disease associations (denoted by A), disease-gene associations (B), and a protein-protein interaction network *G* = (*V*, *E*), where *V* is a set of proteins and *E* is a set of interactions between proteins. The goal of the algorithm is to prioritize all the proteins in *V* with respect to *s*.

Let *F* : *V* → *R* represent a prioritization function; *F* reflects the relevance of *v*  (*v* ∈ *V*) to *s*. *Y* : *V* → [0,1] represent a prior knowledge function, where 1 is assigned to a protein that is known to be related to the disease with respect to *s*, and 0 otherwise. In other words, *Y* is the vector of genes which are known to be causal gene of diseases with respect to symptom. To obtain *Y*, we first analyzed the distribution of similarity between symptom and disease and found that the symptom may have high possibility of relating to a disease when their similarity is above 0.1. Here, we want to choose the diseases which have high possibility to associate with a symptom, so that we could get the related genes to build *Y*. The 10% top ranked disease-symptom relationships with similarities larger than 0.1 are chosen (in our experiment the threshold is 0.57). At last, we selected the ten most related diseases as the diseases corresponding to symptom and its causal genes to build *Y*.

By iterative procedures, the information is transferred between their neighbors, as defined by

(2) $$\begin{array}{c} F^{t}≔\alpha W^{\prime }F^{t-1}+\left(1-\alpha \right)Y, \end{array}$$

where *F* ^1^≔*Y* · *W*′ is a |*V*| × |*V*| matrix which is a normalized form of *W* (described below) and *F* and *Y* are viewed here as vectors of size |*V*|. The details on the inference of *F* in PRINCE algorithm could be found [18]. The parameter *α* ∈ (0,1) weighs the relative importance of these constraints with respect to one another. Here *α* is set to be 0.9 as suggested in the PRINCE algorithm that the appropriate values of *α* could be above 0.5 with fast convergence and 0.9 gets the comparative highest performance [18].

### 3.4. Evaluation Methods

We use Human Phenotype Ontology (HPO) [27] as the benchmark data to evaluate the results. HPO was manually curated from OMIM records and constructed with the goal of covering all phenotypic abnormalities that are commonly encountered in human monogenic diseases [28]. In this study we use the T184 (Sign or Symptom) semantic type of UMLS [29] to filter the phenotype terms and construct a subset of HPO phenotypes (349 records), after filtering the phenotype-genotype associations with focusing on symptoms results in 7,262 symptom-gene records and 1,275 related genes. To deal with the issue of HPO having different symptom terms from MeSH, we used UMLS to map HPO symptom terms to MeSH. We finally obtained 3,418 symptom-gene records with 139 symptoms and 937 genes, which were used for evaluation. Although HPO contains high-quality data on phenotype ontology and genotype-phenotype (mainly on diseases and disorders) associations, the data is rather incomplete and still lack many well-known symptom-gene associations. We evaluated the symptom-gene prediction results by three approaches: (1) compare our rank list with the genes in HPO and calculated recall and AUC [30], (2) compare our result with random case, and (3) evaluate the random chosen results by recent published literatures.

## 4. Results

We extracted 125,226 symptom-disease associations with 322 symptoms and 4,219 diseases from PubMed bibliographic records and calculated the cosine similarity between symptoms and diseases. We constructed 94,536 protein-protein interactions with 14,221 proteins and integrated 28,336 disease-gene associations (shown in Table 1).

**Figure 3:**
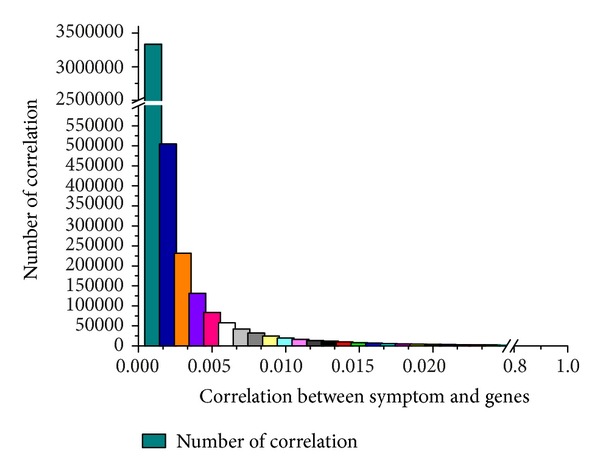
The distribution of correlation between symptom and genes.

The protein-protein interactions were assigned 1 if they are correlated. We used these scores to construct the adjacency matrix *W*. As a result, we obtained totally 4,211,956 symptom-gene associations between 290 symptoms and 14,221 genes with correlation values bigger than zero. The distribution of correlation between symptoms and genes is depicted in Figure 3. It is noted that 83% of the correlations are <0.001, and only about 0.24% are distributed on the range of bigger than 0.01. We consider that the genes with correlation scores bigger than 0.01 have higher possibility than most of the genes (i.e., 83% genes). Therefore, these genes with correlation scores higher than 0.01 are considered to be the potential genes related to symptoms in this study.

**Figure 4:**
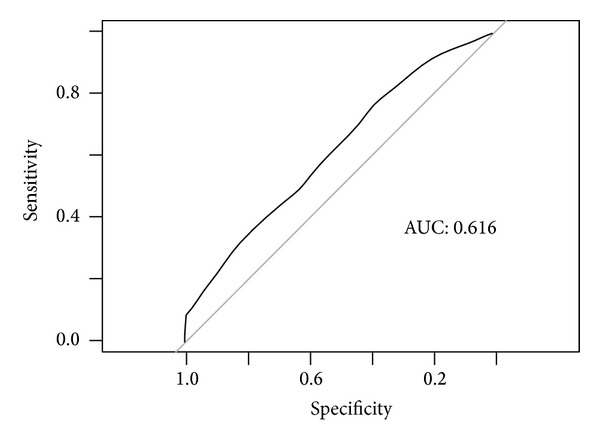
ROC curve to assess prediction performance.

Using the HPO benchmark data, we quantify the accuracy of the prediction by comparing the predicted gene list of symptoms with that of the benchmark data. The area under the ROC curves (AUC) of the proposed method is 0.616 (Figure 4).

In order to evaluate the effectiveness of the gene ranking, we also compared the result with random prediction case. We calculate the quantity of genes contained in HPO on the top of our gene list (*P* < 0.05) by comparing with the average quantity of randomly selected the same number of genes. It is noted that the number of true positive candidate genes is 10-fold of the random prediction, with the best case being 249-fold of the random prediction. We take symptom* Muscle Cramp* as an example to compare our result with random case. Given 27 genes in HPO, there are 10 genes included in the top 251 genes (*P* < 0.05) of our candidate genes list. Randomly choosing 251 genes among all the genes (14,221 genes), the possibility of each gene being causing gene is 0.0018986 (27/14,221, we have the hypothesis that the genes in HPO are all causing genes). The expected number of genes in HPO is 0.477 (0.0018986∗251); that is, there is on average 0.477 true causing genes in HPO gene list if 251 genes are randomly selected. So the number of true positive candidate genes is approximately 20-fold (10/0.477) over the random prediction.

To demonstrate the effectiveness of this method, we listed the suggested genes of headache and hemiplegia for instance. Through the analysis of the distribution of all the scores of symptom related genes, we found that most scores (95% in average) are in very low values (i.e., 0.01) with some exceptions of having much larger scores than these row values.  Table 2 shows the top 46 ranked genes of the 13,966 genes whose correlation scores are greater than 0.01 with respect to the symptom of headache. We found that TNF and EDNRA are the causing genes for headache as listed in HPO. (the Italic font in Table 2, recall is 6.25% of the 32 genes). Several other genes related to headache in HPO including ENG (rank 52th), ACVRL1 (rank 65th), TGFB1 (rank 74th), VHL (rank 269th), COL4A1 (rank 563th), NF2 (rank 1520th), TTR (rank 2270th), MSX2 (rank 2622th), FGFR2 (rank 2636th), PGK1 (rank 2773th), FAM123B (rank 3002th), SH2B3 (rank 3994th), LRP5 (rank 4286th), NOTCH3 (rank 4386th), SDHB (rank 5618th), and CACNA1A (rank 5855th) are ranked in the top 50%.

We were aware that the HPO is an incomplete database. To have a more comprehensive evaluation on the prediction result, we manually searched the literature in PubMed for the symptom-gene associations. Among the top 10 genes of our list, we found that five additional genes CALCA, TGFBR2, ESR1, KCNK18, and MTHFR (bold font in Table 2) are all considered to be related to headache in recent published literatures [31–34], although they are not recognized in the HPO database. As a result, we recognized totally 7 possible causing genes (CALCA, TGFBR2, TNF, ESR1, EDNRA, KCNK18, and MTHFR) of headache in the top 10 genes.

**Figure 5:**
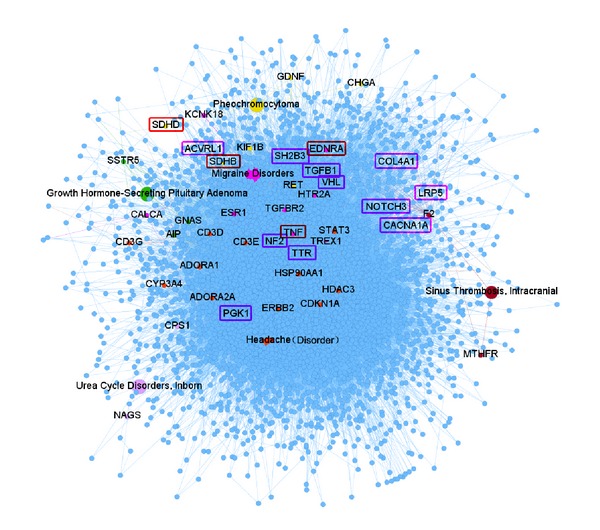
The subset of protein-protein interaction with respect to headache.

**Figure 6:**
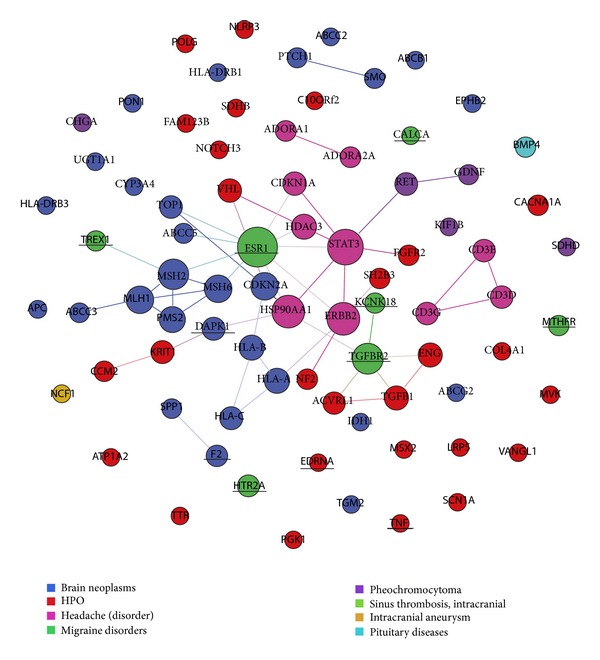
The direct relationship among genes connected with diseases relevant to headache symptom and genes in HPO.

The relationship between symptoms and diseases is complicated. Some symptoms would be more particularly manifested in several diseases than others. This kind of clinical association would have its underlying molecular mechanisms. To explore the interactions of the related genes of symptoms and diseases in the context of PPI network, we show a subset of protein-protein interactions with respect to headache in Figure 5, which is constructed by the genes connected with 6 diseases related to headache directly. In Figure 5, genes connected with the same diseases are marked in the same colors. We found that 15 genes of 32 genes in HPO (marked in box) in our subnet are the causal genes of diseases or locate on their shortest path. It is possible that the causal genes of a disease, which holds the symptom as particular phenotype, would be the related genes for symptom (marked in pink box), or the candidate genes for symptom would possibly locate on the shortest paths of these genes of the diseases, which have the related symptoms as general phenotypes (marked in red box). To have more clear view of the relationships between the candidate genes of symptoms and the casual genes of the diseases holding the corresponding symptoms as particular manifestations, we also constructed a network to show the direct relationships among the causing genes of diseases related to headache and the genes in HPO (Figure 6, genes in HPO are marked in red and genes connected with different diseases are marked with different colors). The genes, CALCA, TGFBR2, TNF, ESR1, EDNRA, MTHFR, and so forth, of our top 10 rank list (mentioned above) are marked with underline. We found that the candidate genes with high scores of headache symptom are the causal genes of the diseases, which regard headache as distinct symptom, such as migraine. It is possible that the causing genes of diseases with respect to the distinct symptoms would also be related to their corresponding symptoms.

Table 3 lists the 83 top ranked genes with respect to hemiplegia with correlation greater than 0.01. In the causing genes of hemiplegia in HPO, four genes, namely, COL4A1, CACNA1A, ATP1A2, and SCN1A, are all found in the top 83 candidate genes (recall is 66.7%) except for the gene DOCK8 which is ranked 6667th in whole list of 14,221 genes. However, we found no related publications on indicating the relationships between the 8 genes (except for the 2 genes included in HPO) of the top 10 genes and hemiplegia after manually searching the PubMed literatures.

## 5. Discussion

As a kind of established clinical manifestations in TCM clinical, symptoms provide key information for the classification of the state of human disease and personalized herb treatment. Symptoms are essentially objective although the observation and description of symptoms incorporate subjective factors like human sense and language. Therefore, investigation of the underlying molecular mechanisms of symptoms is more feasible than TCM syndrome. Through integrating disease-symptom associations and multiple phenotype-genotype data sources, this paper proposes a network inference method to predict the candidate gene list for symptoms. Like similar work for disease gene predictions [35, 36], the rank list of symptom-related candidate genes can promote the discovery of molecular mechanisms of symptoms and thereafter draw the picture of connection between symptoms and genes with respect to diseases. Evaluation shows the effectiveness of the method in identifying genes related to symptoms. Like the predicted genes of headache, more predicted genes could be further investigated to understand the medical insights, which would ultimately support the researchers to confirm the causal genes of symptoms in laboratory study.

It is necessary to mention that this paper is intended to introduce the proposed integrated network framework for predicting the symptom candidate genes. Several aspects related to the method could be improved in future work. Firstly, a carefully curated and evaluated database needs to be established for benchmark data set. Currently, although HPO provides a start point, more effects are needed to obtain high quality symptom-gene databases. While this database is curated, it would offer reliable benchmark platform to evaluations and possible supervision for machine learning methods. On the other hand, due to the complicated confounders involved in symptom-disease relation detection from biomedical literatures, a comprehensive database on disease-symptom relationships would be also very helpful. Secondly, because the similarities between diseases and symptoms indicate different degree of correlations, the similarities between symptoms and diseases could be systematically utilized to improve the iterative computing procedures of random walk related network inference methods. Thirdly, it is highly valuable to investigate the molecular correlations between symptoms and diseases to detect the molecular patterns connecting these two phenotype entities. When some network characteristics underlying the connection are discovered, it would give guideline framework for the development of symptom-gene prediction methods.

## Figures and Tables

**Table 1:** The result of phenotype-genotype data integration.

Number of symptoms	Number of diseases	Number of proteins
322	4,219	14,221

Number of symptom-disease associations	Number of disease-gene associations	Number of protein-protein interactions

125,226	28,336	94,536

**Table 2:** The top 46 rank list of genes predicted with respect to headache.

Number	Symbol	Correlation	Number	Symbol	Correlation
1	**CALCA**	0.12828040612489	26	PROC	0.01319213392192
2	F2	0.12201692087326	27	TGFBR1	0.01314425880888
3	**TGFBR2**	0.12033672267710	28	F2RL3	0.01305473906526
4	*TNF *	0.11489921901213	29	MRPL28	0.01303120114399
5	**ESR1**	0.11394663869964	30	RP1-90J20.6	0.01292643824309
6	**KCNK18**	0.11171558541349	31	NDUFB10	0.01268903908242
7	HTR2A	0.10968115391880	32	GRK6	0.01178478736965
8	*EDNRA *	0.10800919199361	33	BBS4	0.01167226005919
9	TREX1	0.10295846965404	34	THBD	0.01165325302184
10	**MTHFR**	0.10278770151638	35	RAMP3	0.01155667772559
11	CALCRL	0.02849332330671	36	HSP90B3P	0.01154759481953
12	RAMP2	0.02819497378019	37	ATP2B4	0.01147183196121
13	RAMP1	0.02816780187933	38	GGCX	0.01145977743914
14	EDN3	0.02015371056218	39	BBS1	0.01135926399377
15	NAA38	0.01897112176120	40	NME3	0.01105404047118
16	GNA11	0.01715273254725	41	F9	0.01092877796805
17	PROZ	0.01677771421601	42	AKR7A2	0.01081719314504
18	NRD1	0.01533854606373	43	TCTEX1D4	0.01078978137476
19	EDN1	0.01522967177446	44	GP5	0.01063032851227
20	NT5DC3	0.01477167543805	45	SERPINA5	0.01041954082501
21	PDIK1L	0.01477167543805	46	PROS1	0.01034320224653
22	PON2	0.01413140552619			
23	SLC31A2	0.01392606686194			
24	RP11-9H12.2	0.01392606686194			
25	ATP2B2	0.01342703847737			

**Table 3:** The rank of candidate genes with respect to hemiplegia.

Number	Gene symbol	Correlation	Number	Gene symbol	Correlation
1	HMOX1	0.1332389455434	46	CACNB4	0.0132782710879
2	MMP9	0.1287040668111	47	KLK6	0.0131043482021
3	*COL4A1 *	0.1243046971781	48	CXCL6	0.0130491204825
4	SERPIND1	0.1195621966536	49	CXCL1	0.0130308002686
5	PLAT	0.1133487392385	50	TGFBI	0.0129966667251
6	OFD1	0.1121152624575	51	MMP26	0.0128881745922
7	CDKN1A	0.1107796508251	52	BMP3	0.0128183446929
8	STAT3	0.1106360535810	53	UFD1L	0.0126019281191
9	HDAC3	0.1104973436194	54	KISS1	0.0124874609423
10	*CACNA1A *	0.1072041148709	55	LCN2	0.0123446041858
11	PGK1	0.1041298451985	56	CXCL5	0.0122282204837
12	TREX1	0.1033025393792	57	HAPLN1	0.0121753323548
13	MTHFR	0.1028354996936	58	CTSG	0.0121249443517
14	*ATP1A2 *	0.1023789578124	59	SERPINI1	0.0120388399124
15	*SCN1A *	0.1019561553063	60	CABP1	0.0120280578478
16	INPP5E	0.1005153381688	61	CD93	0.0120005018879
17	BLVRB	0.0399295586077	62	COL16A1	0.0119332179348
18	CTA-286B10.6	0.0241333799518	63	PRSS2	0.0119006841698
19	POR	0.0229706000329	64	COL1A1	0.0118755109423
20	COL4A2	0.0203674650216	65	IGHG1	0.0117128695292
21	NAA38	0.0192928165139	66	THBS3	0.0115158569766
22	THBS2	0.0186100092167	67	TFPI	0.0115052630961
23	SAA4	0.0176962732383	68	DCN	0.0112824650786
24	F2	0.0172012123620	69	UBC	0.0110403727935
25	CACNB1	0.0165565034731	70	MMP2	0.0109667286026
26	COL4A4	0.0164204509392	71	COL7A1	0.0109021629899
27	FN1	0.0161953766962	72	LAMA1	0.0106705480427
28	TPT1	0.0159187987328	73	YWHAG	0.0106543097610
29	COL4A3	0.0159043555825	74	IGHA1	0.0104865314738
30	SERPINE2	0.0158807849607	75	RP11-157P1.6	0.0103829026309
31	HABP2	0.0155572576434	76	FAM190B	0.0103401713298
32	COL4A6	0.0154901122445	77	PZP	0.0103015667226
33	COL4A5	0.0153811800445	78	BTC	0.0102664672842
34	SAA2	0.0151416061199	79	NID2	0.0102327719152
35	XXyac-YX65C7_A.1	0.0148026704536	80	TF	0.0101538081025
36	PLG	0.0144321310724	81	RP11-417O11.1	0.0101229813573
37	MATN2	0.0144194792723	82	SERPINA5	0.0101166365026
38	OSM	0.0142128473336	83	NID1	0.0100899609704
39	SNTA1	0.0142116180479			
40	RECK	0.0140016715074			
41	FBLN2	0.0137418115855			
42	COCH	0.0137176242361			
43	MMP10	0.0135697895912			
44	ELANE	0.0134913849548			
45	THBS1	0.0133469773733			

## References

[1] Jiang M, Lu C, Zhang C (2012). Syndrome differentiation in modern research of traditional Chinese medicine. *Journal of Ethnopharmacology*.

[2] Osler W (2005). The principles and practice of medicine: designed for the use of practitioners and students of medicine. *Journal of the American Medical Association*.

[3] Lupski JR, Stankiewicz P (2005). Genomic disorders: molecular mechanisms for rearrangements and conveyed phenotypes. *PLoS Genetics*.

[4] Guo Z, Yu S, Guan Y (2012). Molecular mechanisms of same TCM syndrome for different diseases and different TCM syndrome for same disease in chronic hepatitis B and liver cirrhosis. *Evidence-Based Complementary and Alternative Medicine*.

[5] Reubi JC (2003). Peptide receptors as molecular targets for cancer diagnosis and therapy. *Endocrine Reviews*.

[6] Jiang M, Chen T, Feng H (2013). Serum metabolic signatures of four types of human arthritis. *Journal of Proteomic Research*.

[7] Wu Z, Zhou X, Liu B, Chen J Text mining for finding functional community of related genes using TCM knowledge.

[8] Wheeler DL, Barrett T, Benson DA (2007). Database resources of the national center for biotechnology information. *Nucleic Acids Research*.

[9] Hamosh A, Scott AF, Amberger JS, Bocchini CA, McKusick VA (2005). Online Mendelian Inheritance in Man (OMIM), a knowledgebase of human genes and genetic disorders. *Nucleic Acids Research*.

[10] Cline MS, Smoot M, Cerami E (2007). Integration of biological networks and gene expression data using Cytoscape. *Nature Protocols*.

[11] Rzhetsky A, Iossifov I, Koike T (2004). GeneWays: a system for extracting, analyzing, visualizing, and integrating molecular pathway data. *Journal of Biomedical Informatics*.

[12] Szklarczyk D, Franceschini A, Kuhn M (2011). The STRING database in 2011: functional interaction networks of proteins, globally integrated and scored. *Nucleic Acids Research*.

[13] Barabási A-L, Gulbahce N, Loscalzo J (2011). Network medicine: a network-based approach to human disease. *Nature Reviews Genetics*.

[14] Östlund G, Lindskog M, Sonnhammer ELL (2010). Network-based identification of novel cancer genes. *Molecular and Cellular Proteomics*.

[15] Navlakha S, Kingsford C (2010). The power of protein interaction networks for associating genes with diseases. *Bioinformatics*.

[16] Oti M, Snel B, Huynen MA, Brunner HG (2006). Predicting disease genes using protein-protein interactions. *Journal of Medical Genetics*.

[17] Moreau Y, Tranchevent LC (2012). Computational tools for prioritizing candidate genes: boosting disease gene discovery. *Nature Reviews Genetics*.

[18] Vanunu O, Magger O, Ruppin E, Shlomi T, Sharan R (2010). Associating genes and protein complexes with disease via network propagation. *PLoS Computational Biology*.

[19] Mattingly CJ, Rosenstein MC, Colby GT, Forrest JN, Boyer JL (2006). The Comparative Toxicogenomics Database (CTD): a resource for comparative toxicological studies. *Journal of Experimental Zoology A: Comparative Experimental Biology*.

[20] Hewett M, Oliver DE, Rubin DL (2002). PharmGKB: the pharmacogenetics knowledge base. *Nucleic Acids Research*.

[21] Aguilera M, Arias B, Wichers M (2009). Early adversity and 5-HTT/BDNF genes: new evidence of gene-environment interactions on depressive symptoms in a general population. *Psychological Medicine*.

[22] Costigan M, Woolf CJ (2000). Pain: molecular mechanisms. *Journal of Pain*.

[23] Jeunemaitre X, Soubrier F, Kotelevtsev YV (1992). Molecular basis of human hypertension: role of angiotensinogen. *Cell*.

[24] Zhou X, Menche J, Barabasi AL, Sharma A (2014). Human symptom disease network. *Nature Communications*.

[25] Lu Z (2011). PubMed and beyond: a survey of web tools for searching biomedical literature. *Database*.

[26] Vanunu O, Sharan R A propagation based algorithm for inferring gene-disease associations.

[27] Robinson PN, Köhler S, Bauer S, Seelow D, Horn D, Mundlos S (2008). The human phenotype ontology: a tool for annotating and analyzing human hereditary disease. *American Journal of Human Genetics*.

[28] Robinson PN, Mundlos S (2010). The human phenotype ontology. *Clinical Genetics*.

[29] Bodenreider O (2004). The Unified Medical Language System (UMLS): integrating biomedical terminology. *Nucleic Acids Research*.

[30] Lobo JM, Jiménez-valverde A, Real R (2008). AUC: a misleading measure of the performance of predictive distribution models. *Global Ecology and Biogeography*.

[31] Kaunisto MA, Kallela M, Hämäläinen E (2006). Testing of variants of the MTHFR and ESR1 genes in 1798 Finnish individuals fails to confirm the association with migraine with aura. *Cephalalgia*.

[32] Guldiken B, Sipahi T, Remziye T (2013). Calcitonin gene related Peptide gene polymorphism in migraine patients. *The Canadian Journal of Neurological Sciences*.

[33] Freilinger T, Anttila V, de Vries B (2012). Genome-wide association analysis identifies susceptibility loci for migraine without aura. *Nature Genetics*.

[34] Rainero I, Rubino E, Paemeleire K (2013). Genes and primary headaches: discovering new potential therapeutic targets. *The Journal of Headache and Pain*.

[35] Freudenberg J, Propping P (2002). A similarity-based method for genome-wide prediction of disease-relevant human genes. *Bioinformatics*.

[36] George RA, Liu JY, Feng LL, Bryson-Richardson RJ, Fatkin D, Wouters MA (2006). Analysis of protein sequence and interaction data for candidate disease gene prediction. *Nucleic Acids Research*.

